# A Promoter Region Polymorphism in *PDCD-1* Gene Is Associated with Risk of Rheumatoid Arthritis in the Han Chinese Population of Southeastern China

**DOI:** 10.1155/2014/247637

**Published:** 2014-04-03

**Authors:** CuiPing Liu, JueAn Jiang, Li Gao, XiaoHan Hu, FengMing Wang, Yu Shen, GeHua Yu, ZuoTao Zhao, XueGuang Zhang

**Affiliations:** ^1^Jiangsu Institute of Clinical Immunology, The First Affiliated Hospital of Soochow University, Suzhou 215006, China; ^2^Medical Biotechnology Institute, Soochow University, Suzhou 215006, China; ^3^Wuxi Red Cross Blood Center, Wuxi 214021, China; ^4^Testing Center, Center for Disease Prevention and Control, Changzhou 213000, China; ^5^Department of Dermatology, First Hospital, Peking University, Beijing 100034, China

## Abstract

*Objective*. Programmed cell death 1 (PD-1) induces negative signals to T cells during interaction with its ligands and is therefore a candidate gene in the development of autoimmune diseases such as rheumatoid arthritis (RA). Herein, we investigate the association of *PDCD-1* polymorphisms with the risk of RA among Chinese patients and healthy controls. *Methods*. Using the PCR-direct sequencing analysis, 4 *PDCD-1* SNPs (rs36084323, rs11568821, rs2227982, and rs2227981) were genotyped in 320 RA patients and 309 matched healthy controls. Expression of PD-1 was determined in peripheral blood lymphocytes by flow cytometry and quantitative real-time reverse transcriptase polymerase chain reaction. *Results*. We observed that the GG genotype of rs36084323 was associated with a increased risk for developing RA (OR 1.70, 95% 1.11–2.61, *P* = 0.049). Patients carrying G/G genotype displayed an increased mRNA level of PD-1 (*P* = 0.04) compared with A/A genotype and healthy controls. Meanwhile, patients homozygous for rs36084323 had induced basal PD-1 expression on activated CD4+ T cells. *Conclusion*. The *PDCD-1* polymorphism rs36084323 was significantly associated with RA risk in Han Chinese population. This SNP, which effectively influenced the expression of PD-1, may be a biomarker of early diagnosis of RA and a suitable indicator of utilizing PD-1 inhibitor for treatment of RA.

## 1. Introduction

Rheumatoid arthritis (RA) is a common chronic inflammatory autoimmune disease characterized by significant disability and early mortality, which affects ~1% of adult population worldwide [1, 2]. It is generally accepted that RA is a complex autoimmune disorder, characterized by a chronic T-cell response that evaded normal control mechanisms [3, 4]. Therefore, the genes involved in the regulation of T-cell responses may be primary determinants of susceptibility to RA.

Programmed death-1 (PD-1, also called CD279) is a novel costimulatory member of B7/CD28 family, which is inducibly expressed on CD4+ T cells, CD8+ T cells, natural killer T cells, B cells, and activated monocytes [5]. PD-1 receptor has two ligands: PD-L1 (also known as B7-H1 or CD274) and PD-L2 (also called B7-DC or CD273). PD-L1 is expressed on T cells, B cells, dendritic cells (DC), macrophages, and some tumor cells and is further upregulated upon activation. PD-1 engagement by PD-L1 dephosphorylates proximal signaling molecules and augments PTEN expression, inhibiting PI3K and AKT activation [6, 7]. The critical role of PD-1 in immune regulation is highlighted by gene disruption studies demonstrating strain-specific autoimmune phenotypes [8, 9]. In addition, genetic studies revealed that there is an association between* PDCD-1* gene polymorphism and susceptibility to autoimmune diseases, such as systemic lupus erythematosus (SLE) [10, 11], rheumatoid arthritis [12, 13], multiple sclerosis [14], and diabetes mellitus [15, 16].

There is mounting evidence that PD-1 is linked to human autoimmunity. In view of the pivotal role of PD-1/PD-L pathway in autoimmn immunology, it is worth considering of* PDCD*-*1* functional SNPs,* PDCD-1 *−606A/G (rs36084323, also known as PD1.1) in promoter,* PDCD-1 *+7146A/G (rs11568821, also known as PD1.3) in intron 4, and* PDCD-1 *+7625G/A (rs2227982, also known as PD1.9) and* PDCD-1 *+7786G/C (rs2227981, also known as PD1.5) in exon 5 as prospective candidates for individuals susceptibility to RA in the Han Chinese population. Therefore, in the present study, we sought to determine the association between functional genetic variations in the* PDCD*-*1* gene and RA risk in a Chinese population in mainland.

## 2. Materials and Methods

### 2.1. Patients and Controls

This study was approved by the Ethics Committee of Soochow University and all subjects gave informed consent for the genetic analyses. A total of 320 unrelated Chinese RA patients were recruited from the Outpatient Departments of Rheumatology in the First and the Third Affiliated Hospital of Soochow University. They were composed of 72 men and 248 women, whose mean age was 55.3 years (SD = ±12.6). Individual patients with RA were diagnosed according to the diagnosis criteria established by the American College of Rheumatology and the disease severity of individual patients was evaluated using the disease activity score 28 (DAS28) [1]. A total of 20 patients with new-onset RA (<6 months of disease duration) were recruited for expression of PD-1 protein on activated T cells. Individual RA patients were excluded if she/he received treatment with DMARDs, corticosteroids, or immunosuppressive for any reason during the past 6 months or had other chronic inflammatory and autoimmune diseases, such as diabetes, multiple sclerosis, inflammatory bowel disease, metabolic syndrome, hypertension, cardiovascular diseases, cancer, or recent infection. The controls were gender, age, and ethnically matched unrelated healthy people obtained from the checkup population in the above two hospitals (Table 1).

### 2.2. DNA Extraction and Polymorphism Genotyping

Peripheral venous blood samples of 2 mL were drawn from each individual by standard venepuncture and stored at −20°C. Genomic DNA was isolated from peripheral blood leucocytes by standard procedures. The reference sequence is the human* PDCD-1 *sequence (GeneBank accession number AF363458). The* PDCD-1 *−606A/G (rs36084323) in promoter,* PDCD-1 *+7146A/G (rs11568821) in intron 4, and* PDCD-1* +7625G/A (rs2227982) and* PDCD-1 *+7786G/C (rs2227981) in exon 5 polymorphisms were determined by direct sequencing in an Applied Biosystems sequencer (ABI Prism, Model 3100, Avant).

### 2.3. RNA Isolation and Quantitative Real-Time Reverse Transcriptase Polymerase Chain Reaction

Total RNA from PBMC of 30 RA patients and 24 healthy controls were extracted using Trizol reagent (Invitrogen, Carlsbad, CA) according to the manufacturer's protocol. Total RNA (1 *μ*g) was used for cDNA synthesis with oligodT primers (Invitrogen, Karlsruhe, Germany) and superscript II reverse transcriptase (Takara Bio, Shiga, Japan). PCR was performed using PCR Master (Roche, Mannheim, Germany) with the following primers: for PD-1 mRNA, 5′-CTCAGGGTGACAGAGAGAAG-3′ (forward) and 5′-GACACCAACCACCAGGGTTT-3′ (reverse) and for GAPDH, 5′-GTGAAGGTCGGAGTCAACG-3′ (forward) and 5′-TGAGGTCAATGAAGGGG-TC-3′ (reverse). Fold changes were normalised based on GAPDH expression, and each assay was conducted in a 96-well ABI 7900HT real-time PCR system (Applied Biosystems, Foster City, CA, USA). This procedure was performed in triplicate.

### 2.4. Antibodies and Flow Cytometry

All antibodies were mouse anti-human monoclonal antibodies (mAb). PE-Cy5-conjugated anti-CD4 (OKT4) were from Beckman Coulter (Miami, FL). PE-conjugated anti-PD-1 (MIH4), fluorescein isothiocyanate (FITC)-conjugated anti-CD25 (M-A251), anti-CD69 (FN50), and anti-HLA-DR (G46-6) were from BD PharMingen (Franklin Lakes, NJ). PE- or PE-Cy5-conjugated IgG1 (679.1Mc7) (Beckman Coulter), PE-conjugated IgG2a (eBM2a; eBio-science), and FITC-conjugated IgG1 (P3) (eBioscience) were used as IgG isotype controls. PBMCs (0.5 × 10^6^) were incubated in wash buffer (PBS/2.5% fetal bovine serum [FBS]) with appropriate amounts of mAb on ice for 30 minutes. Cells were washed and were immediately analyzed on an EPICS XL-MCL flow cytometer (Beckman Coulter).

### 2.5. Statistics Analysis

The deviation from Hardy-Weinberg equilibrium (HWE) was examined in controls by the  *χ* ^2^ test. The following statistical analyses were performed using SNPstats software (Availability: http://bioinfo.iconcologia.net/SNPstats) [17]. Based on the logistic regression method, the case-control association of genotypes was tested and the odds ratios (OR) and 95% confidence intervals (95% CI) were given. *D*′ and *r* ^2^ were calculated to evaluate the magnitude of LD. Haplotype frequencies were estimated using the EM algorithm coded into the haplo.stats package (http://mayoresearch.mayo.edu/mayo/research/biostat/schaid.cfm). The association analysis of haplotypes was similar to that of genotypes with logistic regression, and results were shown as OR and 95% CI. The most frequent haplotype was automatically selected as the reference category and rare haplotypes were pooled together in a group. The log-additive inheritance model was assumed by default. The significance level of all these tests was 0.05. The nonparametric Mann-Whitney *U* test was used for comparisons among groups with small or unequal sample sizes. Results were expressed as the mean ± SEM, and 2-tailed *P* values less than 0.05 were considered significant.

## 3. Results

### 3.1. Single Nucleotide Polymorphism Analysis

A total of four SNPs were successfully genotyped in 320 RA patients and 309 healthy controls.  Table 2 shows the allele and genotype distribution of these four SNPs. *P* values for Hardy-Weinberg proportions of the SNPs are shown in Table 2 as well. For all four SNPs, the genotypic distribution in controls conformed to HWE. Among the four SNPs, the genotype and allele distributions of rs36084323 differed significantly between RA patients and controls (*P* < 0.05). When logistic regression was used for association analysis after modeling the SNPs' effects as additive, dominant, or recessive, rs36084323 showed significant difference in codominant (OR, 1.70; 95% CI, 1.11–2.61), recessive (OR, 1.50; 95% CI, 1.05–2.14), and log-additive (OR, 1.30; 95% CI, 1.05–1.61) models (Table 3). The log-additive model was accepted as the best inheritance model because it showed the smallest Akaike information criterion value (869.4). The rs11568821 nucleotide is not polymorphic among Chinese population. The other two SNPs showed no association with RA in all 5 inheritance models (data not shown).

### 3.2. Linkage Disequilibrium and Haplotypes Association Analysis

Because rs11568821 was nonpolymorphic in our population, it was excluded from the haplotype construction and LD analysis. Pairwise LD between the SNPs of the* PDCD-1* (rs36084323, rs2227982, and rs2227981) was calculated for the cases and controls in the Han Chinese. We found strong LD (*D*′ > 0.75) between some pairs of markers in the* PDCD-1* gene including rs36084323/rs2227982 (*D*′ = 0.7729) and rs2227982/rs2227981 (*D*′ = 0.7841).

The association analysis of the haplotypes with RA was similar to that of genotypes by logistic regression (Table 4). We found that the haplotype of rs36084323/rs2227982/rs2227981 showed significant association with the disease (*P* = 0.00015); ACT and ACC haplotypes were less frequently observed in cases than in controls (OR: 0.23, 95% CI: 0.10–0.53; OR: 0.32, 95% CI: 0.11–0.96, resp.), indicating that these two haplotypes act as protective phenotype in RA. The association analysis of the haplotypes was adjusted by sex too.

### 3.3. Association between rs36084323 Polymorphism and PD-1 mRNA Expression

**Figure 1:**
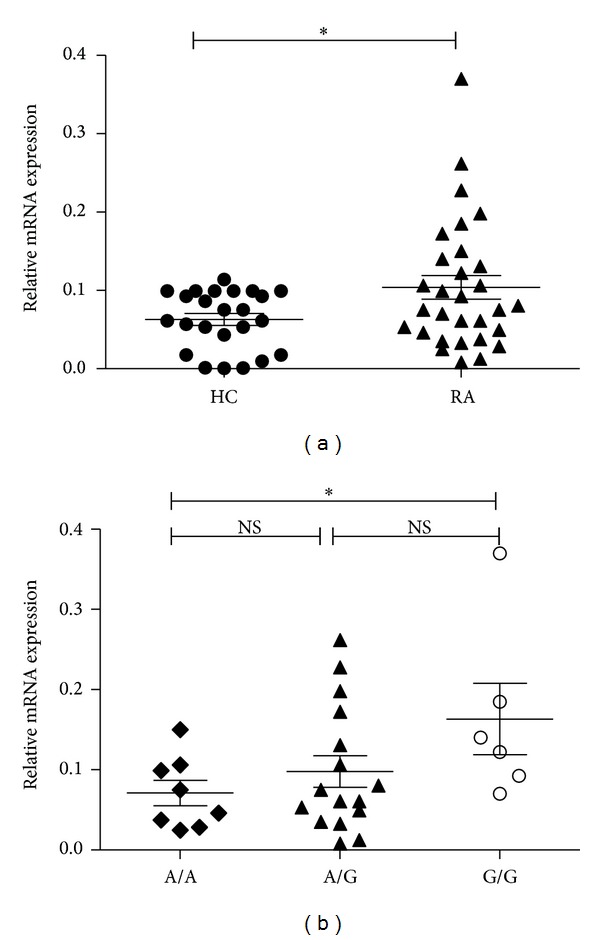
Analysis of PD-1 mRNA expression levels in RA patients with different genotypes of rs36084323. (a) Increased levels of PD-1 mRNA levels in PBMC of RA (*n* = 30) patients, as compared with those from healthy controls (HC) (*n* = 24) (*P* = 0.027). (b) The patients with GG genotype exhibited higher PD-1mRNA expression levels than those with AA genotype (*P* = 0.049). Horizontal bars indicate the mean ± SD, * = *P* < 0.05.

To evaluate the association between rs36084323 polymorphism and RA, we examined whether or not the rs36084323 polymorphism was associated with an altered PD-1 mRNA expression. Significant difference was observed in the relative PD-1 mRNA expression level between patients with GG and AA genotypes (Figures 1(a) and 1(b)). We concluded that* PDCD-1* polymorphisms were associated with RA, and the GG genotype and G allele of rs36084323 that associated with increased PD-1 mRNA expression might be involved in RA development in Han Chinese population.

### 3.4. Upregulated Expression of PD-1 on Activated CD4+T Cells in RA Patients Was Associated with rs36084323

**Figure 2:**
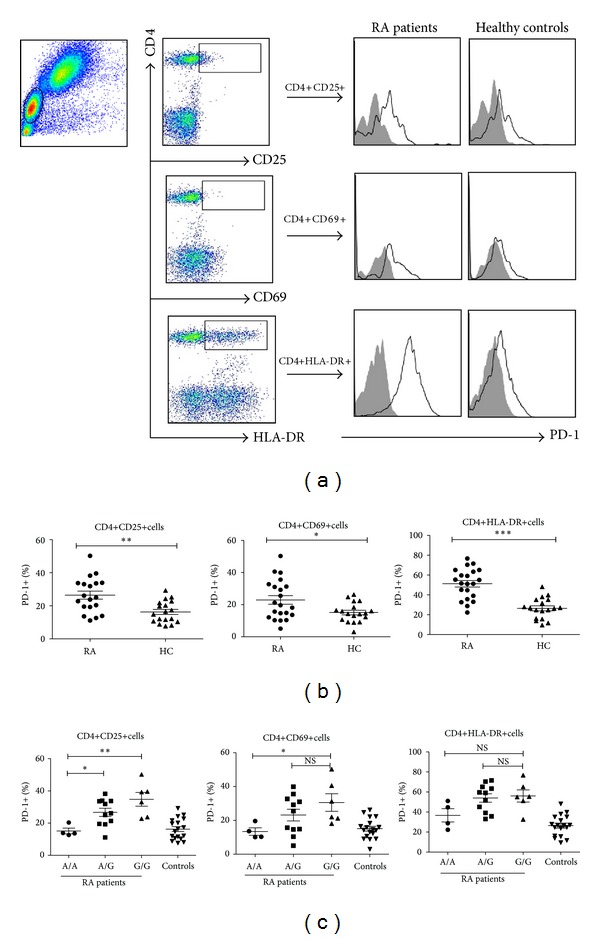
Increased basal programmed death 1 (PD-1) expression on activated CD4+ T cells in RA patients. (a) A representative flow cytometry analysis of PD-1 expression on CD4+ T cells in RA patients and healthy controls is shown. (b) Upregulated expression of PD-1 on CD4+CD25+, CD4+CD69+, and CD4+HLA-DR+ T cells from RA patients, as compared with those from healthy controls. (c) RA patients homozygous for the rs36084323 SNP (G/G) have significantly increased percentages of PD-1+activated CD4+CD25+, CD4+CD69+, and CD4+HLA-DR+ T cells compared with healthy controls and rs36084323 A/A RA patients (* = *P* < 0.05, ** = *P* < 0.01 by Mann-Whitney *U* test). Horizontal bars indicate the mean ± SD.

We next examined the expression of PD-1 on PBMCs in RA patients. Flow cytometry analyses of PBMC samples from 20 RA patients demonstrated the upregulated PD-1 expression on CD4+CD25+T cells (26.44 ± 2.43 versus 16.3 ± 1.54, *P* = 0.0014), CD4+CD69+T cells (22.95 ± 2.68 versus 15.10 ± 1.432, *P* = 0.0185), and CD4+HLA-DR +T cells (51.26 ± 3.31 versus 26.46 ± 2.469, *P* < 0.0001) compared with healthy controls (Figures 2(a) and 2(b)). Although we found no difference between wild-type (G/G) and heterozygous (A/G) patients, the 4 patients who were homozygous for the SNP rs36084323 (A/A) had minimal PD-1 expression on freshly isolated CD4+T cells, including the activated CD25+ and CD69+ T-cell subsets (Figure 2(c)). Collectively, these results suggest that the rs36084323 polymorphism confers increased basal PD-1 expression at early-to-intermediate stages of CD4+ T-cell activation and that this is associated with increased risk of RA.

## 4. Discussion

Rheumatoid arthritis is a chronic inflammatory disease that may involve extra-articular organs in addition to joints. Genetic and environmental factors are related to the pathogenesis of RA [1, 2]. PD-1 has been implicated as a critical pathway for tolerance since the first discovery of spontaneous autoimmune disease in PD-1 knockout mice. Studies using the NOD mouse model for spontaneous type I diabetes have shown that PD-1 not only is critical during the early phases of T-cell activation and expansion, but also plays a critical role in regulating T-cell effector functions and T-cell tolerance at late time points in peripheral tissue sites [16–19]. Strategies to selectively target the PD-1 pathway using antagonists have been effective in CIA model. Administration of PD-L1-Ig significantly ameliorated autoimmunity as assessed by clinical arthritis score and histology in the joints [20]. Similarly, PDL-1.Fc treatment ameliorated the severity of CIA and reduced joint swelling as well as antigen-specific T-cell proliferative responses [21]. PD-1 knockout mice have increased susceptibility and develop severe arthritis following immunization with type II collagen [21]. Collectively, these models demonstrate the importance of PD-1 in the pathogenesis of RA.

Greater than 30 single nucleotide polymorphisms (SNPs) have currently been identified in the human* PDCD-1* gene, and several SNPs have been linked to development of autoimmunity in various populations [10]. Understanding how these SNPs impact PD-1 functions in human cells would be important for exploiting this pathway clinically. In our research, we selected four potentially functional polymorphisms (The* PDCD-1 *−606A/G (rs rs36084323) in promoter,* PDCD-1 *+7146A/G (rs11568821) in intron 4, and* PDCD-1 *+7625G/A (rs2227982) and* PDCD-1 *+7786G/C (rs2227981) in exon 5) of* PDCD-1* gene and identified the association between the four polymorphisms and the risk of RA in Southeastern Chinese population.

A region of* PDCD-1* intron 4 was described as an enhancer-like structure containing binding sites for several transcription factors [22]. ^P^7146 G/A (PD-1.3) SNP, a regulatory SNP located in intron 4, showed to be involved in susceptibility to RA in Swedish, European American, and Mexican families and in sporadic cases. However, we found that this nucleotide is not polymorphic in our Chinese people, and this was also verified among other Chinese populations [13, 23, 24].

As it is known, mutations in the promoter region may influence the engagement between sequence motifs and transcription factors and further disrupt the activation of gene and the initiation of transcription [25]. Accordingly, as a polymorphism located in the promoter of* PDCD-1* gene, rs36084323 may also affect the transcription and activation of* PDCD-1 *gene, thereby influencing the development of RA. These promoter polymorphisms have been considered risk factors for RA. One group observed that the frequency of SNP PD-1.1 (rs36084323) A allele is a risk allele in Italian RA patients [26]. Another group observed that the AA genotype of SNP PD-1.1 was associated with a decreased risk for developing RA in Hong Kong Chinese RA patients [13]. Previous reports also have shown that PD-1.1 was associated with risk of sporadic breast cancer [27]. We observed reduced frequencies of both the AA genotype and the A allele in RA patients compared with controls and a compensatory increase of the GG genotype in RA patients, which indicated that AA genotype might be a protective factor to decrease the risk of RA. This was consistent with the results from Hong Kong Chinese population [13]. Hence, rs36084323 may be a functional polymorphism which can influence the risk of diseases.

PD-1 is thought to be important for the “fine tuning” of lymphocyte activation at the level of synovial tissue, considering the wide pattern of expression of one of its ligands, PDL-1, in activated endothelial and epithelial cells [17, 28–30]. Furthermore, CD4+PD-1+ T cells accumulate as unique anergic cells in rheumatoid arthritis synovial fluid [31]. It is also reported that the negative costimulatory PD-1/PDL-1 pathway regulates peripheral T-cell responses in both human and murine RA [21]. In this study we found upregulated PD-1 expression on CD4+CD25+T cells, CD4+CD69+T cells, and CD4+HLA-DR+T cells compared with healthy controls. We also found that patients carrying G/G genotype displayed an increased mRNA level of PD-1 and increased basal PD-1 expression on activated CD4+ T cells. We concluded that PD-1 polymorphisms were associated with RA, and the GG genotype and G allele of rs36084323 that associated with induced PD-1 mRNA expression and PD-1 expression might be risk factor in RA development in the Han Chinese population.

In conclusion, the present study provides strong evidence that rs36084323 functional polymorphisms may contribute to the risk of RA. This SNP, which effectively influenced the expression of PD-1, may be a biomarker of early diagnosis of RA and a suitable indicator of utilizing PD-1 inhibitor for treatment of RA. However, our results were obtained from a sample-sized sample, and therefore this is a preliminary conclusion. Validation by a larger prospective study from a more diverse ethnic population is needed to confirm these findings.

## Figures and Tables

**Table 1:** Characteristics of RA patients and controls.

Characteristic	RA patients	Controls	*P* value
Total number	320	309	
Female	248 (77.5)	240 (77.7)	>0.05^a^
Male	72 (22.5)	69 (22.3)	
Age, mean ± SD years	55.3 ± 12.6	52.9 ± 10.7	>0.05^b^
Duration of disease, years	12.1 ± 8.0		
RF+^c^	261 (81.6)		
RF−^c^	59 (19.4)		
Anti-CCP+^c^	244 (76.4)		
Anti-CCP−^c^	76 (23.6)		

Values are numbers (%); RA: rheumatoid arthritis; RF: rheumatoid factor; anti-CCP: anticyclic citrullinated peptide.

^a^ *P* value calculated by Pearson chi-square test (all frequency >0.05) or Fisher's exact test (any frequency <0.05).

^b^ *P* value calculated by Student's *t*-test.

^c^Clinical data were not available for some cases.

**Table 2:** The 4 SNPs of *PDCD-1* gene investigated in the cases (*n* = 320) and controls (*n* = 309).

SNP	dbSNP ID and position		Frequency (%)							HWE
			Allele		*P*	Genotype			*P*	*P*
1	rs36084323		A	G		A/A	A/G	G/G		
	promoter (−606A/G)	Cases	47.2	52.8		24.7	45.1	30.3		
		Controls	54.1	45.9	0.014*	31.1	46.5	22.5	0.049*	0.12

2	rs11568821		A	G		A/A	A/G	G/G		
	Intron 4 (+7146A/G)	Cases	—	100		—	—	100		
		Controls	—	100		—	—	100	—	—

3	rs2227982		C	T		C/C	C/T	T/T		
	Eoxn 5 (+7625G/A)	Cases	51.5	48.5		24.8	53.5	21.8		
		Controls	52.5	47.5	0.74	29.6	45.8	24.6	0.19	0.21

4	rs2227981		C	T		C/C	C/T	T/T		
	Exon 5 (+7786G/C)	Cases	73.2	26.8		54.8	36.8	8.4		
		Controls	68.0	32.0	0.06	47.0	42.2	10.9	0.18	0.65

HWE indicates Hardy-Weinberg equilibrium; *P < 0.05.

**Table 3:** The association analysis of rs36084323 with RA (adjusted by sex) by using logistic regression.

Model	Genotype	Cases (%)	Controls (%)	OR (95% CI)	*P* value	AIC	BIC
Codominant	A/A	24.7	31.1	1	0.049*	871.3	884.6
	A/G	45.1	46.5	1.22 (0.84–1.78)			
	G/G	30.3	22.5	1.70 (1.11–2.61)			

Dominant	A/A	24.7	31.1	1	0.073	872.1	881
	A/G-G/G	75.3	68.9	1.38 (0.97–1.96)			

Recessive	A/A-A/G	69.7	77.5	1	0.026*	870.3	879.2
	G/G	30.3	22.5	1.50 (1.05–2.14)			

Overdominant	A/A-G/G	54.9	53.5	1	0.73	875.2	884
	A/G	45.1	46.5	0.95 (0.69–1.29)			

Log-additive	—	—	—	1.30 (1.05–1.61)	0.015*	869.4	878.3

OR: odds ratio; 95% CI: 95% confidence intervals; AIC: Akaike's information criterion; BIC: Bayesian information criterion; *P < 0.05.

**Table 4:** Estimated haplotype frequencies and the association analysis with RA.

Haplotype	Sequence	Cases	Controls	Total	OR (95% CI)	*P* value
1	ATC	0.4108	0.4061	0.4081	1.00	—
2	GCC	0.2583	0.1919	0.2248	1.28 (0.94–1.73)	0.11
3	GCT	0.2127	0.2066	0.2098	0.98 (0.72–1.32)	0.87
4	GTC	0.0523	0.0462	0.0497	1.04 (0.53–2.02)	0.91
5	ACT	0.019	0.0821	0.0463	0.23 (0.10–0.53)	6*e* − 04*
6	ACC	0.0138	0.0420	0.0280	0.32 (0.11–0.96)	0.043*
7	ATT	0.0269	0.0139	0.0210	1.61 (0.52–5.02)	0.41
^a^Rare	—	—	—	0.0081	1.55 (0.27–8.96)	0.68
	Global haplotype association *P* value: 0.0015*					

OR: odds ratio; 95% CI: 95% confidence intervals.*P < 0.05.

^a^Rare haplotypes were pooled together in a group.
